# Semantic segmentation of urban environments: Leveraging U-Net deep learning model for cityscape image analysis

**DOI:** 10.1371/journal.pone.0300767

**Published:** 2024-04-05

**Authors:** T. S. Arulananth, P. G. Kuppusamy, Ramesh Kumar Ayyasamy, Saadat M. Alhashmi, M. Mahalakshmi, K. Vasanth, P. Chinnasamy

**Affiliations:** 1 Department of Electronics and Communication Engineering, MLR Institute of Technology, Hyderabad, India; 2 Department of Electronics and Communication Engineering, Siddharth Institute of Engineering & Technology, Puttur, Andhrapradesh, India; 3 Faculty of Information and Communication Technology, Universiti Tunku Abdul Rahman, Kampar, Perak, Malaysia; 4 College of Computing and Informatics, University of Sharjah, Sharjah, UAE; 5 Department of Networking and Communications, SRM Institute of Science & Technology, College of Engineering and Technology, Kattankulathur, Tamil Nadu, India; 6 Department of Electronics and Communication Engineering, Chaitanya Bharathi Institute of Technology, Hyderabad, Telangana, India; 7 Department of Computer Science and Engineering, MLR Institute of Technology, Hyderabad, Telangana, India; Sichuan University, CHINA

## Abstract

Semantic segmentation of cityscapes via deep learning is an essential and game-changing research topic that offers a more nuanced comprehension of urban landscapes. Deep learning techniques tackle urban complexity and diversity, which unlocks a broad range of applications. These include urban planning, transportation management, autonomous driving, and smart city efforts. Through rich context and insights, semantic segmentation helps decision-makers and stakeholders make educated decisions for sustainable and effective urban development. This study investigates an in-depth exploration of cityscape image segmentation using the U-Net deep learning model. The proposed U-Net architecture comprises an encoder and decoder structure. The encoder uses convolutional layers and down sampling to extract hierarchical information from input images. Each down sample step reduces spatial dimensions, and increases feature depth, aiding context acquisition. Batch normalization and dropout layers stabilize models and prevent overfitting during encoding. The decoder reconstructs higher-resolution feature maps using "UpSampling2D" layers. Through extensive experimentation and evaluation of the Cityscapes dataset, this study demonstrates the effectiveness of the U-Net model in achieving state-of-the-art results in image segmentation. The results clearly shown that, the proposed model has high accuracy, mean IOU and mean DICE compared to existing models.

## 1. Introduction

Semantic segmentation of urban landscapes using deep learning has emerged as a central research subject in recent years [1], because of its transformational potential in comprehending complicated cityscapes and enabling a variety of applications. The primary idea behind semantic segmentation is the accurate tagging of each pixel in an image to a particular item or class [2]. This allows for a more nuanced comprehension of how the picture is put together. Semantic segmentation differs from typical image classification in that it does not give a single label to an entire picture. Instead, labels are assigned at the pixel level, which enables a comprehensive and context-rich study of urban settings.

The need for semantic segmentation in the context of Cityscapes emerges [3] from the need to glean useful insights from large-scale urban photographs and films. This necessitates the use of such technology. It is becoming more important to have effective urban planning, transportation management, and public safety measures in place as the population of the globe continues to urbanize at an alarming rate [4–6]. Through the use of semantic segmentation, a variety of features such as roads, buildings, people, automobiles, and plants are able to automatically recognized and classified [7]. This enables more in-depth urban study and thereby useful in making more informed decisions.

The capacity of deep learning to deal with the complexity and diversity of cityscapes [8] is the primary reason for the use of deep learning models in semantic segmentation. Deep learning models, in particular convolutional neural networks (CNNs), have shown very high levels of performance in a variety of image identification tasks, including semantic segmentation [9–11]. Their ability to learn hierarchical and abstract characteristics from data enables them to catch complicated patterns in urban landscapes [12,13] which ultimately leads to segmentation findings that are precise and reliable.

The use of semantic segmentation in cityscapes [14,15] has a wide-ranging and significant range of application scope. It provides urban planners and policymakers with the opportunity to gather useful insights about traffic patterns, pedestrian movement, and the distribution of land use, which helps in the design of efficient transportation systems and urban infrastructure. In addition, effective semantic segmentation may be of considerable assistance to autonomous cars, allowing them to traverse complicated urban surroundings in a safe manner while also making well-informed judgments [16,17]. In addition, semantic segmentation plays an important role in a variety of smart city activities, including waste management, environmental monitoring, and public safety [18], all of which contribute to an improvement in the overall quality of life for those who live in urban areas.

Significant contributions of the proposed system

- The objective is to utilise the U-Net deep learning model to explore cityscape image segmentation.
- The purpose is to utilise an encoder with convolutional layers to extract hierarchical metadata from input images through down-sampling processes.
- The decoder applies up-sampling layers to recreate high-resolution feature maps.
- The performance assessment of the suggested approach is compared to the most advanced segmentation methods available.
- The suggested method attains superior levels of accuracy, mean Intersection over Union (IoU), and mean DICE scores compared to existing methods.

The remaining paper is structured as follows: part 2 details the in-depth analysis of cityscapes using deep learning techniques, and section 3 discusses the suggested approach. Section 4 discusses the efficiency analysis of the suggested approach. In section 5, we finally come to an end.

## 2. Literature survey

Li et al.’s study [19] developed a technique that involves combining numerous baselines or backbone networks, improving object inner consistency and sharpening object boundaries. The proposed technique underwent rigorous evaluation on four notable road scene semantic segmentation benchmarks, namely Cityscapes, CamVid, KITTI, and BDD. The comprehensive testing outcomes demonstrate that the method proposed achieves a new state-of-the-art performance level, while also demonstrating notable efficiency during inference.

Antonin Vobecky et al. [20] synchronized LiDAR and image data were employed to explore the potential of cross-modal unsupervised learning in the context of semantic image segmentation. To contribute to this field, the necessity of devising a novel technique capable of addressing the challenges associated with this type of learning was recognized. The approach relies on an object proposal module that analyses the LiDAR point cloud to provide suggestions for spatially consistent objects, a pivotal element of the methodology. Furthermore, the authors illustrate the alignment of these 3D object recommendations with input images and their effective categorization into semantically relevant pseudo-classes. This achievement is accomplished through the use of both an alignment method and a clustering algorithm. In conclusion, a cross-modal distillation technique is formulated for image semantic segmentation, employing partially labeled image data to train a transformer-based model.

Liu et al. [21] introduced a semantic segmentation network named BFMNet, aiming to address the aforementioned challenges in an efficient and streamlined manner. The authors initiate the process by employing a lightweight bilateral structure to store both semantic and detailed information extracted from images. Subsequently, they incorporate feature interactions during the encoding phase of this structure. Additionally, to enhance the network’s ability to capture information from objects at multiple scales, which is critical for semantic segmentation, a dedicated Multi-Scale Context Aggregation Module (MSCAM) is developed.

Quan Zhou et al [22] offered a unique encoder-decoder framework for semantic segmentation termed the contextual ensemble network (CENet). Within this architectural framework, contextual hints are consolidated through the dense upsampling of convolutional features from deep layers to the shallower deconvolutional layers, all with the ultimate aim of accomplishing semantic segmentation. The suggested CENet is trained to match the resolution of the input picture in terms of end-to-end segmentation, and it enables us to fully investigate contextual information via an ensemble of dense deconvolutions. The authors analyse the performance of the CENet using two semantic segmentation datasets that are quite popular: PASCAL VOC 2012 and CityScapes.

M. Naseer Subhani et al [23] presented a unique strategy for using the semantic segmentation model’s scale-invariance trait for self-supervised domain adaptation. The technique is based on the logical premise that, in general, the semantic labeling should remain the same independent of the size of the object and things (provided context). The authors demonstrate how this restriction is broken in the pictures of the target domain, allowing labels to be transferred across patches of various sizes. They specifically demonstrate that, when scaled-up patches of the target domain are provided, as opposed to when original size pictures are presented, the semantic segmentation model delivers output with high entropy. These scale-invariant samples were taken from the target domain’s most reliable photos. To remove erroneous pseudo-labels, a dynamic class-specific entropy thresholding approach is provided. Additionally, in order to address the issue of class imbalance in self-supervised learning, they additionally introduce the focus loss.

Ying Yang et al. [24] introduced a Context Aggregation Network (CAN) as a dual-branch convolutional neural network. The CAN model was observed to exhibit enhanced prediction accuracy in comparison to prior methodologies, along with substantial reductions in processing overhead. To advance the state-of-the-art methods for efficient semantic segmentation, the proposal was made by the researchers to establish a context branch. This particular branch incorporates streamlined versions of global aggregation and local distribution blocks, adept at capturing both distant and nearby contextual dependencies efficiently.

Haq et al [25], The 3D-CNNHSR model was applied after a methodical hyperspectral pre-processing phase to reduce noise and boost the signal-to-noise ratio. After pre-processing, the Hyperion dataset’s bands have been decreased from a total of 242 categories to 159 categories. For all 159 groups, the super-resolution technique was used. Considering MPSNR, MSE, and MSSIM measurements depending on the Sentinel-2 dataset with a resolution of 10 m, the 3D-CNNHSR’s effectiveness was assessed. For the Hyperion 2015 and Hyperion 2017 SR visuals, the generated model demonstrated a high MPSNR of 58.987 and 58.912, accordingly. In Anul Haq et al [26], Five machine learning strategies were created, one of which, SMOTEDNN, was a unique model designed to tackle identifying indicators of pollution in the atmosphere. Each of the five models makes use of thorough hyperparameter optimization techniques and effective data initial processing. Outstanding functionality was demonstrated by all created models in terms of sensitiveness, specificity, accuracy, and precision. Notably, compared to the other models from the present research and other investigations, the unique model SMOTEDNN demonstrated superior accuracy (99.90%). Eight LSTM layers total—three layers with dropouts and four dense layers—were created in the current study to create the CDLSTM model. Rigid parameter tweaking served as the basis for the optimization of the CDLSTM model. Based on a number of criteria, including R2, MSE, RMSE, MAPE, MAD, and NSE, the constructed CDLSTM model performed admirably. Given its dependability, the created CDLSTM model was probably going to be able to predict future precipitation and temperature values with accuracy by Haq [27]. The UAV imagery-based plant identification with CNN is introduced and analysed with different methods in terms of accuracy in [28,29]. To handle assault and non-attack occurrences within the system, two types were suggested in [30]. The NSL-KDD dataset was used to test the suggested methodologies, and the results showed accuracy scores of 99.34% and 99.13% for binaries and multi-class categorization, correspondingly. A completely automated method for identifying two datasets of brain tumors is described. The first population consists of almost 400,000 images from the MICCAI-RSNA [31].

Xiao et al. [32] proposed a Local Spatial Pixel Adjustment Network (LSPANet), which primarily consists of two pivotal components: a Spatial Pixel Cross-Correlation (SPCC) block and a Dual-Branch Decoding Fusion (DDF) module. The DDF module is engineered to amalgamate diverse information gathered during the encoder stage. It takes inputs from high-level and low-level feature maps derived from various stages and progressively mitigates dissimilarities in their content. The SPCC block leverages the HSPA module to record the spatial correlation between individual pixel values and their adjacent pixels in the horizontal dimension, while the VSPA module performs a similar function in the vertical dimension. This facilitates the assignment of pixel value weights based on their specific spatial positions within the image. The evaluation of LSPANet’s performance is conducted using the Cityscapes and Camvid datasets.

Zhang et al. [33] devised an innovative network known as GPNet, which effectively filters multi-scale data through a gated and paired approach, enabling comprehensive data aggregation. The Gated Pyramid Module (GPM) is custom-designed to integrate low-level and high-level features, yielding receptive fields that are both dense and expansive. The authors of the GPM paper advocated for the establishment of a gated route that efficiently filters extraneous information across various levels of granularity in the datasets. Utilizing contextual information extracted from shallow layers is recommended to guide the deep features via the incorporation of a Cross-Layer Attention Module (CLAM). The edge detection based fast pixel matching and PCA based image segmentation is discussed in [34,35].

In recent years, we have seen substantial advancements in the semantic categorization of urban landscapes using deep learning models such as U-Net. To further enhance the functionality and usefulness of these models, a number of research gaps and issues still need to be resolved. The following are a few of the research gaps that have been found when employing U-Net deep learning models to categorize urban surroundings semantically:

1. The reliability of Various Atmospheric Conditions: A lot of current models, such as U-Net, are susceptible to changes in the weather, lighting, and urban environments. To make these models more resilient and flexible to a range of environmental circumstances, more research is required.
2. Small Object Detection: Small things such as street furniture, pedestrians, and traffic signs are frequently seen in urban environments. Improving U-Net models’ capacity to identify and divide up such tiny items is a crucial task.
3. Morphological Interpretation of 3D Urban Surroundings: A new field of study entails expanding semantic models for segmentation to improve comprehension of 3D urban environments, which include topography and buildings. Combining LiDAR data as well as depth information with U-Net algorithms is an interesting direction.
4. Interpretable Models: The practical deployment of these advancements depends on the development of techniques to improve the interpretability of U-Net and related deep-learning models for participants such as architects and legislators.

By filling in these research gaps, we are introducing the semantic segmentation based on Deep learning-based U-Net model especially for urban contexts will continue to progress and become more accurate, dependable, and useful for a variety of applications in transportation, smart cities, and urban planning.

**Table:** 

Study	Technique Used	Evaluation Metrics	Performance
Li et al. [19]	Combining baselines/backbone networks	Cityscapes, CamVid, KITTI, BDD	State-of-the-art performance, notable efficiency
Antonin Vobecky et al. [20]	Cross-modal unsupervised learning	LiDAR and image data	Transformer-based model
Liu et al. [21]	BFMNet semantic segmentation network	Lightweight bilateral structure, Multi-Scale Context Aggregation Module (MSCAM)	-
Quan Zhou et al. [22]	Contextual ensemble network (CENet)	PASCAL VOC 2012, CityScapes	-
M. Naseer Subhani et al. [23]	Self-supervised domain adaptation	Semantic segmentation model’s scale-invariance trait	Dynamic class-specific entropy thresholding, focus loss
Ying Yang et al. [24]	Context Aggregation Network (CAN)	Dual-branch convolutional neural network	Enhanced prediction accuracy, reduced processing overhead
Haq et al. [25]	3D-CNNHSR	Hyperspectral pre-processing, super-resolution	High MPSNR
Anul Haq et al. [26]	SMOTEDNN model	Machine learning strategies, hyperparameter optimization	Superior accuracy
Haq [27]	CDLSTM	LSTM layers, parameter tweaking	Good performance based on various criteria
[28,29]	CNN	UAV imagery based plant identification with CNN	-
[30]	Assault and non-attack occurrence handling	NSL-KDD dataset	High accuracy scores
[31]	Automated method for identifying brain tumors	MICCAI-RSNA dataset	-
Xiao et al. [32]	Local Spatial Pixel Adjustment Network (LSPANet)	Spatial Pixel Cross-Correlation (SPCC) block, Dual-Branch Decoding Fusion (DDF) module	Cityscapes, Camvid datasets
Zhang et al. [33]	GPNet network	Gated Pyramid Module (GPM), Cross-Layer Attention Module (CLAM)	-
[34,35]	Edge detection, PCA-based image segmentation	PCA Algorithm	-
Proposed	Deep Learning based U-Net	Semantic Segmentation	Mean DICE, Mean IoU, Accuracy

## 3. Proposed model

The preprocessing processes for semantic segmentation, particularly when utilising a U-Net deep learning model for cityscape picture analysis, encompass numerous crucial stages. Max pooling and batch normalisation are essential elements of the U-Net architecture, contributing to feature extraction and enhancing the stability of model training. Now, let’s examine the preprocessing phases, with a particular emphasis on these procedures:

1. Data Collection and Annotation:—Collect a varied dataset of urban environment photos along with appropriate annotations at the pixel level. Verify that the annotations precisely depict the desired categories, such as roads, buildings, pedestrians, and so on.
2. Image resizing and Uniformity:—Adjust the dimensions of the photos to a uniform input size that is appropriate for the U-Net architecture. Ensure consistency by normalising pixel values and harmonising colour spaces throughout the dataset. This phase guarantees that the model is provided with inputs of uniform dimensions during the training process.
3. Normalization:—Standardise pixel values by normalising them to a standardised range. Ensuring speedier convergence during training and reducing the model’s sensitivity to input intensity changes, this stage is crucial. Popular normalisation methods involve rescaling pixel values to a specific range, such as [0, 1], or employing z-score normalisation.
4. Data Enhancement:—Implement data augmentation strategies to enhance the diversity of the dataset. Methods such as stochastic flips, rotations, zooming, and cropping aid in enhancing the model’s ability to generalise across diverse viewpoints and settings.
5. Mask Encrypting:—Transform pixel-level annotations into categorical masks that are compatible with the U-Net output format. Every pixel must be allocated a distinct label that corresponds to its specific class. This phase guarantees that the ground truth annotations are in perfect alignment with the model’s anticipated output.
6. Batch Normalization:—Incorporate batch normalisation layers into the U-Net design. Batch normalisation aids in stabilising and expediting training by standardising the input of every layer across mini-batches. This mitigates the occurrence of internal covariate shift, resulting in reduced sensitivity of the model to the initial weights and learning rates.
7. U-Net Model Setting up:—Set up the U-Net model by integrating max pooling layers for reducing the resolution and batch normalisation layers for ensuring stability. The encoder component of the U-Net employs max pooling to decrease spatial dimensions, capturing hierarchical characteristics. The decoder component utilises up-sampling layers to restore the spatial information.
8. Max Pooling:—Incorporate max pooling layers to reduce the size of feature maps during the encoding process. Max pooling is a technique that captures important features while decreasing the size of the data, allowing the model to concentrate on more meaningful patterns. Ensuring a balance between computational efficiency and feature extraction is of utmost importance in this step.
9. Image Preprocessing:—Implement supplementary preprocessing techniques, such as histogram equalisation or colour space transformations, to amplify significant characteristics in urban scene segmentation. These techniques can enhance the model’s ability to accurately capture fine details in cityscape photos.
10. Data Splitting:—Partition of the dataset into training, validation, and test sets. Make sure that each collection retains an equitable distribution of the many classes seen in urban landscapes.

**Fig 1:**
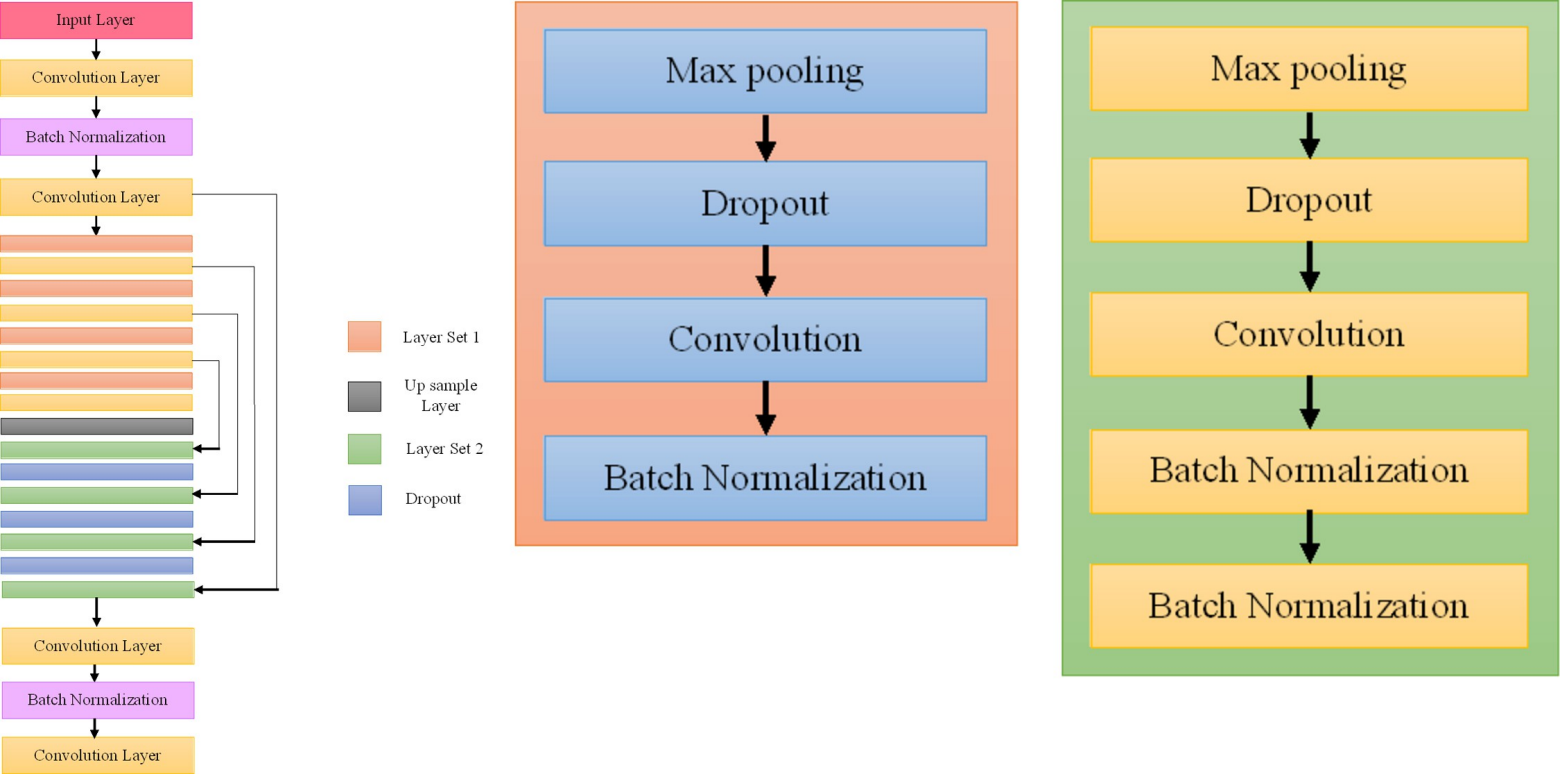
Proposed U-Net architecture. (a) U-Net Model. (b) Layer set 1. (c) Layer set 2.

Fig 1, explain the proposed U-Net architecture. U-Net is a deep learning framework that can be utilized for semantic segmentation tasks. In this model, an image is first segmented into relevant areas, and then each pixel is tagged with a specific label that is associated with a certain category or category of objects. The term UNet is coined owing to the U-shaped architecture of the network. The network comprises two distinct pathways: the encoder, situated on the left side of the U shape, and the decoder, positioned on the right side of the U. The encoder enhances the resolution of feature maps to construct a dense segmentation map with spatial dimensions identical to those of the original image. Conversely, the decoder diminishes spatial dimensions and encompasses the entire image context.

- **Encoder**: In the conventional UNet, the encoder consists of multiple convolutional layers, each of which is accompanied by a rectified linear unit (ReLU) activation function and max-pooling layers. As a result of this chain of operations, the contextual information included within the image is understood while the spatial dimensions are reduced, which ultimately results in a condensed representation of the input.
- **Bottleneck**: A bottleneck layer is located at the very bottom of the U-shaped design. This layer is comprised of numerous convolutional layers, and its purpose is to further refine the features that were learnt in the contracting route.
- **Decoder**: The decoder is made up of a series of upsampling layers, which often use transpose convolutions or interpolation to progressively increase the spatial dimensions of the feature maps. These upsampling layers are arranged in a succession. At each stage of the upsampling process, the associated feature maps from the encoder are brought together and connected to form skip connections. These links are essential because they allow the decoder to access low-level feature information, which in turn makes precise localization throughout the segmentation process easier to achieve.

The last step of the UNet model is a 1x1 convolutional layer that uses a softmax activation function, which is responsible for classification. This layer will provide a probability map for each and every pixel, taking into account all of the classifications. The pixel is then assigned a label based on the category that has the greatest likelihood of occurring at that particular location. The UNet model commonly uses cross-entropy loss function for training. This loss function involves making a comparison between the predicted probability map and the ground truth segmentation map.

### 3.1 Proposed UNet encoder

The encoder in the UNet architecture is used for extracting hierarchical characteristics from the input images. It is made up of a sequence of convolutional layers and downsampling layers, with the goal of gradually decreasing the spatial dimensions while simultaneously increasing the number of dimensions.

- The first downsample starts with a 3x3 convolutional layer and applies 64 filters on the input image tensor. The padding is now set to "same," which indicates that the output will maintain the same spatial dimensions as the input data. The convolutional operation output is then processed through a Rectified Linear Unit (ReLU) activation function, which introduces non-linearity. Next, batch normalization is performed to the output to normalize it. This step contributes to the stability of the training and speeds up convergence. The normalized output is subjected to an additional 3x3 convolutional layer that has 64 filters applied to it. This output, which is referred to as "f2," is saved for use in a residual connection at a later time. Then, a max-pooling layer is applied with a pool size of 2x2 and a stride of 2, which halves the amount of space that is occupied by the spatial dimensions. When using max-pooling, the largest value from each local area is taken, which results in the feature maps being downsampled. During the training process, a dropout layer is implemented with a rate of 0.2 in order to avoid overfitting.
- The second downsample follows a pattern that is quite similar to the first. Following the application of the dropout layer, the output is fed into a 3x3 convolutional layer that has 128 filters and uses ReLU activation. After performing batch normalization and then adding another 3x3 convolutional layer that has 128 filters, the output, which is designated with "f6," is stored for use in a residual connection. Another round of downsampling is performed on the feature maps using max-pooling, and then a dropout layer is added.
- The procedure is repeated once again using a 3x3 convolutional layer that has 256 filters and ReLU activation for the third downsample. Batch normalization is performed, followed by the application of a further 3x3 convolutional layer with 256 filters. The output (which is denoted by "f10") is saved for use in a residual connection, and the feature maps are then downsampled using max-pooling, after which a dropout layer is added.
- A 3x3 convolutional layer with 512 filters and ReLU activation is used for the fourth downsample, which is similar to the previous one. The image then undergoes batch normalization before having a further 3x3 convolutional layer with 512 filters added to it. The output (which is denoted by "f14") is saved for use in a residual connection, and the feature maps are then downsampled using max-pooling, after which a dropout layer is added.
- The last downsample, the fifth, begins with a 3x3 convolutional layer that has 1024 filters and ReLU activation. After that, a batch normalization step is implemented, and finally, a further 3x3 convolutional layer with 1024 filters is added. The encoder’s final output is denoted by the letter "f18" in the output.

During the encoding phase of the UNet architecture, the progressive downsampling operations of the encoder allow the model to acquire more high-level characteristics and context. This enables the model to have a better understanding of the input pictures and to make more accurate predictions during the decoding phase that follows.

**Pseudocode**: UNet encoder

**Input**: Image of size 128, 128

**Output**: Feature map

# First Downsample

f1 = Conv2D(inputs, 64, (3, 3), "same", 1, "relu")

b1 = BatchNormalization(f1)

f2 = Conv2D(b1, 64, (3, 3), "same", 1, "relu")

m3 = MaxPooling2D(f2, (2, 2), 2)

d4 = Dropout(m3, 0.2)

# Second Downsample

f5 = Conv2D(d4, 128, (3, 3), "same", 1, "relu")

b5 = BatchNormalization(f5)

f6 = Conv2D(b5, 128, (3, 3), "same", 1, "relu")

m7 = MaxPooling2D(f6, (2, 2), 2)

d8 = Dropout(m7, 0.2)

# Third Downsample

f9 = Conv2D(d8, 256, (3, 3), "same", 1, "relu")

b9 = BatchNormalization(f9)

f10 = Conv2D(b9, 256, (3, 3), "same", 1, "relu")

m11 = MaxPooling2D(f10, (2, 2), 2)

d12 = Dropout(m11, 0.2)

# Forth Downsample

f13 = Conv2D(d12, 512, (3, 3), "same", 1, "relu")

b13 = BatchNormalization(f13)

f14 = Conv2D(b13, 512, (3, 3), "same", 1, "relu")

m15 = MaxPooling2D(f14, (2, 2), 2)

d16 = Dropout(m15, 0.2)

# Fifth Downsample

f17 = Conv2D(d16, 1024, (3, 3), "same", 1, "relu")

b17 = BatchNormalization(f17)

f18 = Conv2D(b17, 1024, (3, 3), "same", 1, "relu")

### 3.2 Proposed UNet decoder

In the decoder, a series of upsampling layers employing the "UpSampling2D" layer are employed, facilitating the expansion of spatial dimensions within the feature maps. To mitigate overfitting, dropout regularization is subsequently applied, and the upsampled feature maps are merged with the feature maps generated by previous layers. The incorporation of a skip-connection formed by concatenating the encoder’s layers enables the network to blend high-level features with lower-level features. The convolutional layers that use batch normalization are added so that the feature maps may be refined while the upsampling process is taking place.

- During the first step of upsampling, UpSampling2D is used to increase the resolution of the feature maps by a factor of 2. In order to prevent overfitting, a dropout layer is added, and then the upsampled feature maps are concatenated with the feature maps produced from a layer from the encoder (f14). The concatenated tensor is sent through two convolutional layers, each of which has 512 filters.
- During the second upsampling, the procedures from the first phase are repeated and UpSampling2D is used with an additional factor of 2. Dropout is performed once again for the purpose of regularization, and then the upsampled feature maps are concatenated with the feature maps from a layer from the encoder (f10). After that, these concatenated tensors are sent through two convolutional layers, each of which consists of 256 filters.
- In the third phase of upsampling, UpSampling2D is repeated in order to increase the resolution of the feature maps by a factor of 2, and then dropout and concatenation with the feature maps from f6 are performed. After the tensors have been concatenated, they are sent through two convolutional layers, each of which has 128 filters.
- In the fourth phase, UpSampling2D is used which is followed by dropout and concatenation using feature maps from f2. The next step involves applying two convolutional layers, each of which has 64 filters.

The skip connections allow the network to learn from both local and global data, which ultimately leads to improved segmentation performance. During the training phase, the use of dropout helps to regularize the model and prevents overfitting from occurring. Batch normalization, which works by standardizing the activations of the convolutional layers, is another technique that contributes to the stabilization of training.

**Pseudocode**: UNet decoder

**Input**: Feature map

**Output**: Segmented image

# First Upsample

m19 = UpSample(f18, size = (2, 2))

d19 = Dropout(m19, 0.2)

c20 = Concatenate([d19, f14])

f21 = Conv2D(c20, 512, (3, 3), "same", 1, "relu")

b21 = BatchNormalization(f21)

f22 = Conv2D(b21, 512, (3, 3), "same", 1, "relu")

# Second Upsample

m23 = UpSample(f22, size = (2, 2))

d23 = Dropout(m23, 0.2)

c24 = Concatenate([d23, f10])

f25 = Conv2D(c24, 256, (3, 3), "same", 1, "relu")

b25 = BatchNormalization(f25)

f26 = Conv2D(b25, 256, (3, 3), "same", 1, "relu")

# Third Upsample

m27 = UpSample(f26, size = (2, 2))

d27 = Dropout(m27, 0.2)

c28 = Concatenate([d27, f6])

f29 = Conv2D(c28, 128, (3, 3), "same", 1, "relu")

b29 = BatchNormalization(f29)

f30 = Conv2D(b29, 128, (3, 3), "same", 1, "relu")

# Fourth Upsample

m31 = UpSample(f30, size = (2, 2))

d31 = Dropout(m31, 0.2)

c32 = Concatenate([d31, f2])

f33 = Conv2D(c32, 64, (3, 3), "same", 1, "relu")

b33 = BatchNormalization(f33)

f34 = Conv2D(b33, 64, (3, 3), "same", 1, "relu")

## 4. Experimental results

**Fig 2:**
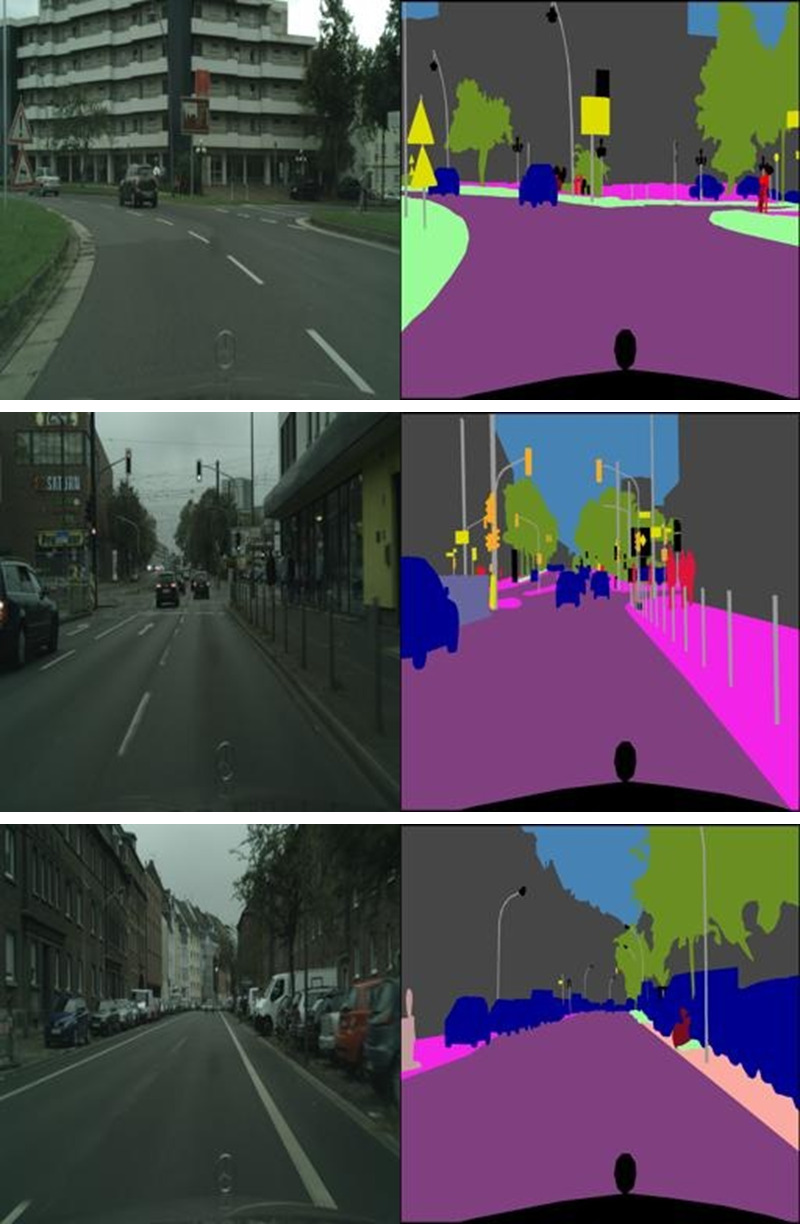
Sample training images in the dataset.

The Cityscapes dataset is made up of movies that were annotated and recorded from moving automobiles across Germany. The dataset contains still photos that were retrieved from the source films, and semantic segmentation labels are supplied with each image in the set. The 3D boundaries annotations encompass all eight semantic classes within the vehicle classification of the Cityscapes dataset, namely car, truck, bus, on rails, motorbike, bicycle, caravan and trailer. Notably, this dataset is held in very high esteem for semantic segmentation tasks, and its quality is such that it is recognized as being among the very finest in this field. The validation subset has 500 images, whereas the training subset has a total of 2975 image files. Each image has a resolution of 256 by 512 pixels and presents a composite view consisting of the original photograph on the left half of the file and the matching annotated image (the result of semantic segmentation) on the right half of the file. The system setup of the proposed method is mentioned in Table 1. The sample dataset images are depicted in Fig 2.

**Table 1:** The proposed model parameter setup.

Hyperparameter	Configurations
Activation function	“softmax”
Optimizer	Adam
Learning rate	0.0001
Batch size	32
Epochs	20–25
Metrics	IoU & mean IoU
Input images size	128 × 128

The use of the U-Net deep learning model in the image segmentation of Cityscapes involves a series of procedures that facilitate the precise segmentation of urban landscapes. This technique proves valuable in several domains such as autonomous driving and urban planning. The data preparation process begins by acquiring the Cityscapes dataset, which consists of photographs accompanied with pixel-level annotations specifically designed for semantic segmentation. The dataset is then partitioned into several sets designated for training, and validation objectives. In order to achieve consistency, the photographs undergo resizing to a standardized dimension, and the pixel values are normalized within an appropriate range.

The U-Net architecture, which has been selected for its effectiveness in image segmentation, represents the second phase. The system consists of an encoder pathway designed for the purpose of extracting features, as well as a decoder pathway that aims to preserve context. The encoder utilizes convolutional and pooling layers to collect hierarchical features, whilst the decoder utilizes up-sampling and convolutional layers to restore spatial resolution and create segmentations. Significantly, the inclusion of skip-connections in the design allows for the merging of feature maps derived from both the encoder and decoder, hence improving the accuracy of segmentation.

The categorical cross-entropy is a widely used loss function for the job at hand. The loss function assesses the disparity between anticipated and actual segmentation’s, hence directing the model’s iterative improvement. After the building of the model, the next step is compilation, which involves the use of an optimizer such as Adam and the selection of an appropriate loss function. During the training phase, the model is provided with training images and its predicted segmentations are compared to the ground truth annotations in order to calculate the loss. The model’s weights are adjusted iteratively by the optimizer in order to minimize the loss and improve the accuracy of segmentation.

**Fig 3:**
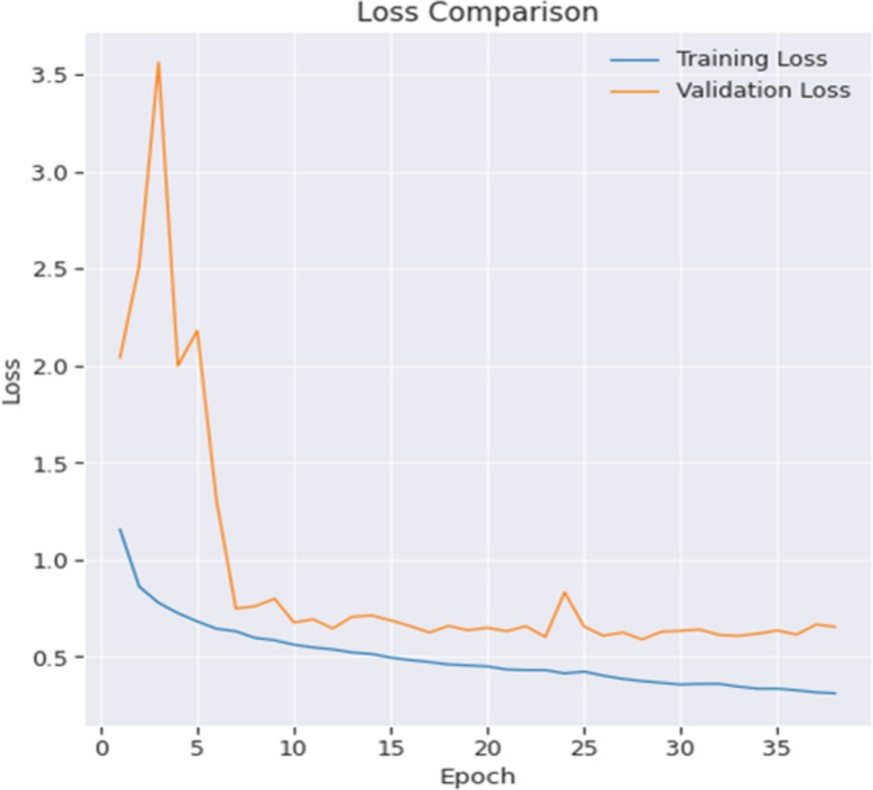
Loss comparison of proposed model.

The process of validation is of utmost importance throughout the training phase, as it involves regularly evaluating the model’s performance on a separate validation set. This evaluation serves the purpose of monitoring progress and minimizing the potential dangers associated with overfitting. After the completion of the training process, the model undergoes evaluation using the test set in order to assess its capacity to generalize and perform well on new and previously unknown data. If necessary, further processes like as noise reduction and segmentation refining are implemented in order to enhance the quality of the findings. The loss comparison of the proposed model is depicted in Fig 3.

A complete evaluation of the model’s performance has been conducted via an investigation into the efficacy of various loss functions. The study involves a comprehensive investigation of several loss functions, each designed to address certain elements of the model’s objective. The aim of this study is to evaluate the influence of various loss functions on the predictive accuracy and feature segmentation capabilities of the model. This comparative analysis aims to determine the best appropriate methodology for maximizing the performance of the model, hence improving its capacity to provide accurate and contextually meaningful predictions, specifically in the domain of picture segmentation. Graphical illustrations of the training loss and validation loss in Fig 3 have been produced to visually portray the advancement of the model’s learning process. The aforementioned charts provide valuable information about the model’s adaptability to the training data and its capacity to generalize to unknown data during the validation process. Through the examination of the trends and patterns shown in these loss curves, one may get a more comprehensive comprehension of the model’s convergence and performance. This, in turn, facilitates the evaluation of its training dynamics and overall efficacy.

**Fig 4:**
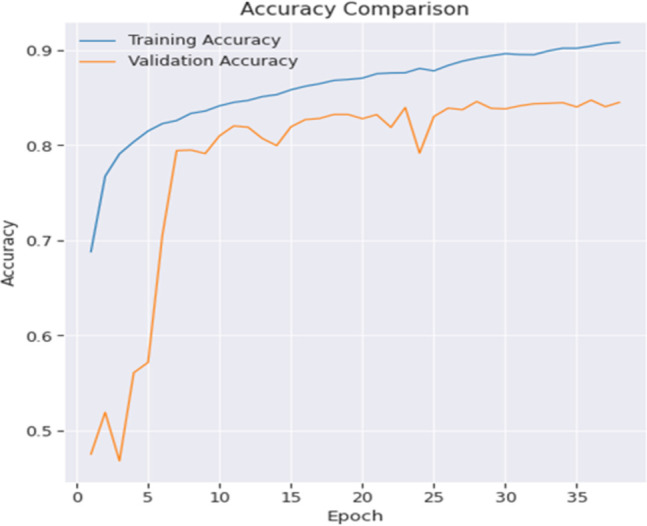
Accuracy comparison of proposed model.

To illustrate the model’s learning process, graphical representations of the training accuracy and validation accuracy have been depicted in Fig 4. The graphs provide a comprehensive representation of the model’s performance in accurately identifying data in both the training and validation stages. Examining the patterns and variations in these accuracy curves provides significant information regarding the model’s capacity to acquire knowledge from the training dataset and extrapolate its predictions to new, unfamiliar data. The use of visualizations aids in the evaluation of the training dynamics of the model and its efficacy in attaining precise predictions across diverse datasets.

**Fig 5:**
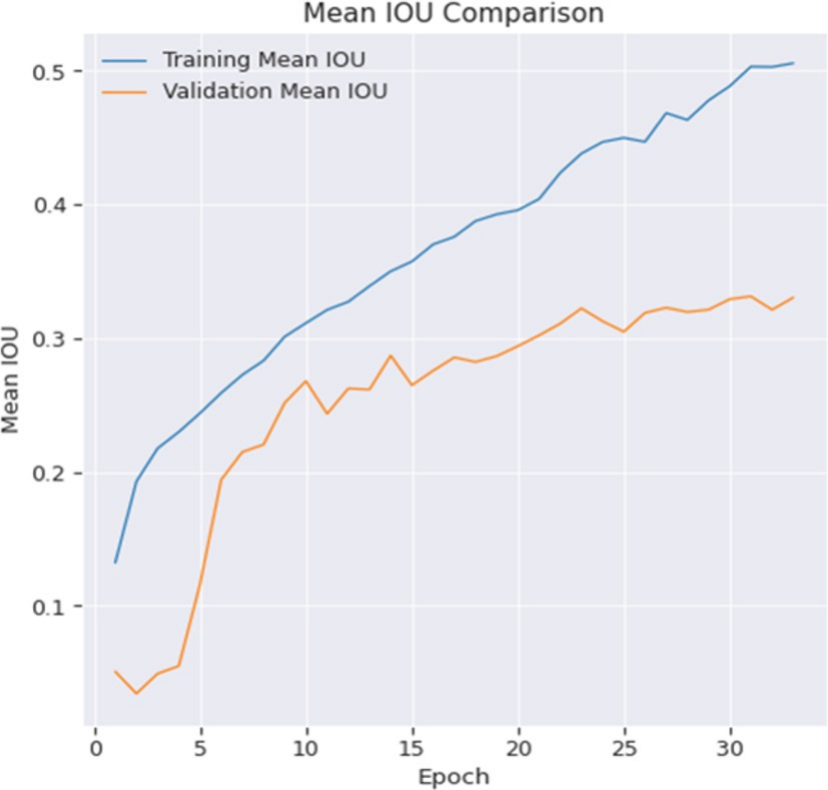
Mean IoU comparison of the proposed model.

**Fig 6:**
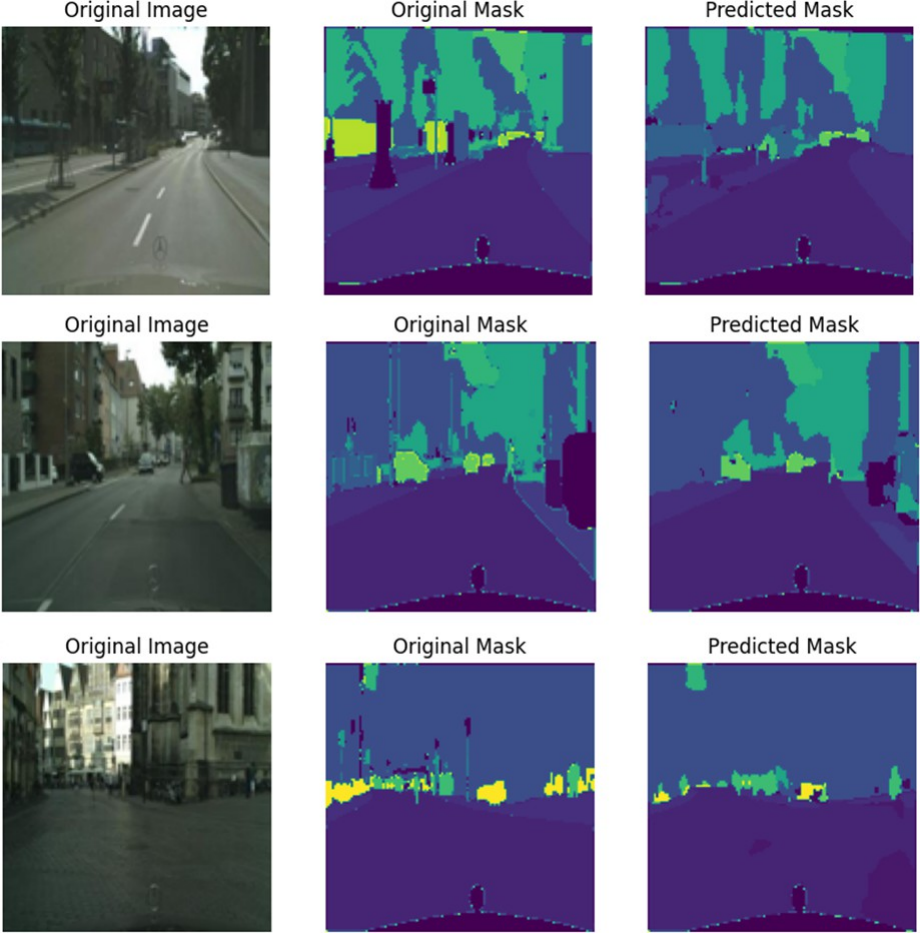
Segmented results of proposed model.

To illustrate the model’s semantic segmentation performance, Training and Validation Mean Intersection over Union (MoU) graphics have been depicted in Fig 5. These graphs demonstrate the model’s picture pixel and area classification accuracy. Trends and variations in these MoU curves reveal the model’s ability to capture object boundaries and spatial connections throughout training and validation. These metrics help analyze the model’s segmentation effectiveness and capacity to deliver accurate and consistent results. The segmented output results are shown in Fig 6.

It is evident that after undergoing 35 epochs of training, the model has not reached its optimal performance level. Since the layers are initialized from scratch, it is expected that a higher level of patience will provide better results. The training and validation performance for most of the metrics exhibit a high degree of similarity, with the exception of the IOU score. It is evident from the Mean IOU plot that the model begins to exhibit overfitting tendencies after around 20–25 epochs, as shown via a side-by-side comparison with accuracy and loss.

The segmented outcomes of the proposed model are shown in Fig 6 and it is a structured layout consisting of three columns: the initial picture, the initial mask, and the predicted mask. This presentation facilitates a juxtaposition of the input picture, the ground truth mask, and the mask created by the model’s predictions for the purpose of comparison. The organization of this setup enables a visual examination of the model’s ability to reliably identify and categorize items in the photos. This assists in assessing the model’s segmentation skills and identifying areas that need improvement. In conclusion, the U-Net model that has undergone training may be effectively used to segment novel photos or urban landscapes, hence providing precise predictions at the pixel level for every class. The use of this extensive methodology guarantees that the U-Net deep learning model, when utilized for Cityscapes picture segmentation, provides a thorough and meaningful comprehension of urban landscapes, so making a valuable contribution to the improvement of urban planning and its associated applications.

**Fig 7:**
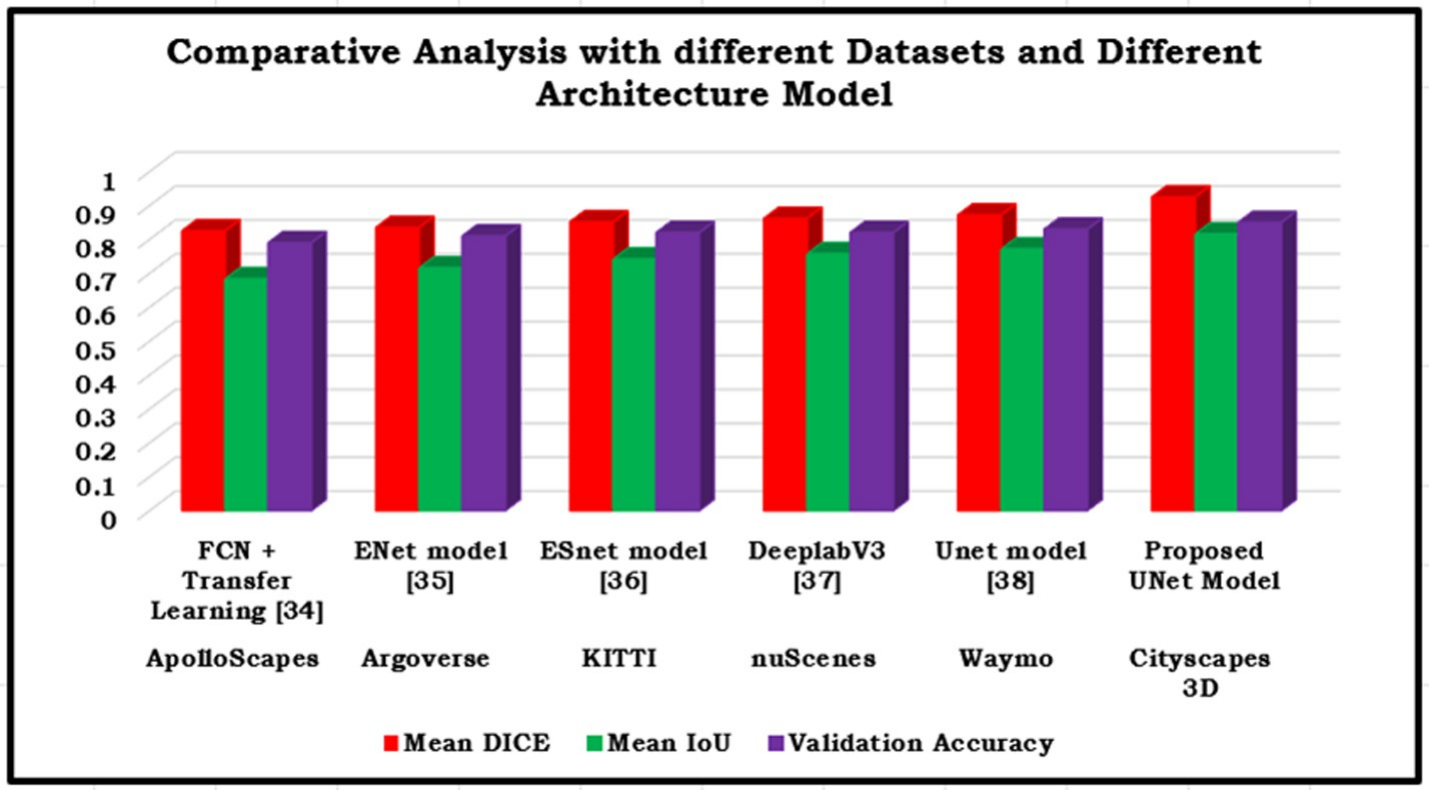
Schematic representation of the performance analysis with various model and various datasets.

The Table 2 presents a thorough comparison of several models, each designed specifically for unique tasks related to data categorization and segmentation for various datasets. The primary parameter being assessed is the performance of the models during the validation phase, whereby their ability to effectively categorize and segment data is prominently shown. The model known as "FCN + Transfer Learning" has commendable performance, achieving a validation accuracy of 0.79. This performance is attributed to the use of transfer learning, which allows the model to leverage its inherent advantages. The "ENet Model" demonstrates exceptional performance with a validation accuracy of 0.81, which is well recognized for its notable computational economy. The "Proposed UNet Model" stands out as the frontrunner, with the greatest validation accuracy of 0.85. This result highlights its remarkable ability to accurately classify and segment data. This comparative study offers useful insights into the relative merits of different models, assisting in the decision-making process for selecting the best appropriate model for certain tasks in the realm of data analysis and segmentation as shown in Fig 7.

**Table 2:** Performance analysis with various model.

Data Sets	Model Name	Mean DICE	Mean IoU	Validation Accuracy
ApolloScapes	FCN + Transfer Learning [36]	0.825	0.684	0.79
Argoverse	ENet model [37]	0.835	0.716	0.81
KITTI	ESnet model [38]	0.8515	0.7422	0.82
nuScenes	DeeplabV3 [39]	0.8617	0.758	0.82
Waymo	Unet model [40]	0.8719	0.772	0.83
Cityscapes 3D	**Proposed UNet Model**	**0.925**	**0.8162**	**0.85**

Compared to Unet Model [40], which has more layers and parameters and is therefore larger and increasingly complicated, the proposed has a model that is smaller in size. More intricate structures and characteristics in the data are made possible by the larger model size. Nevertheless, because of its higher sophistication and feature count, UNet Model [40] requires more time to train. According to the design, machine configuration, and kind of dataset, these frameworks’ precise computing costs and performance may change. The variety of features and time costs associated with our model are displayed in Table 3.

**Table 3:** The computational analysis of the proposed model.

Types	Total Parameters	Training Parameters	Non-trained Parameters	Time(s)
Unet Model [40]	26.435.207	27.708.435	124.234	121, 422
Proposed	77.048.467	76.467.792	315.812	120, 472

However, "Our Model" received better segmentation scores—0.968 for identification of real time images and 0.974 for other identification—than the other models. This indicates that in terms of precisely segmenting the urban area image regions, "Our Model" fared better than the "Reference" framework.

## 5. Limitations and future scope of the proposed method

1. Reliability and Generalization: Although U-Net and other deep learning models have produced some outstanding results, they may still have difficulty correctly segmenting items in intricate urban landscapes, particularly when dealing with uncommon objects or scenarios or in bad weather. Enhancing the accuracy and generality poses a significant problem.
2. Data Annotation: It takes a lot of money and effort to produce thorough and accurate ground truth descriptions for metropolitan landscapes. One major problem is to scale up the acquisition of high-quality labelled data.
3. Real-time Computation: Real-time processing is necessary for many urban applications, including autonomous driving. The real-time processing demands of these applications might not always be satisfied by U-Net and related models because to their computational complexity.

Future Scope:

1. Better designs: Work is being done to create models that are lightweight and other neural network designs for semantic segmentation that are more effective and economical. Another intriguing direction is to combine U-Net with different architectures and methodologies.
2. Multi-modal Fusion: The precision and resilience of urban assessment of the environment can be greatly enhanced by including multi-modal information from sensors in the categorization procedure.
3. Real-time Optimization: To make algorithms based on deep learning appropriate for applications that require real-time data, the investigation into hardware speed, model enlargement, and effective inference approaches is necessary.
4. Domain Adaptation: It will be important to conduct research to find ways to modify models trained in one urban context to function well in another, even when the data contains domain shifts.
5. Robustness and Safety: Retaining categorization models’ resilience under varied environmental circumstances and resolving safety issues in self-driving vehicles continue to be formidable obstacles.

## 6. Discussion

The U-Net architecture is a suggested paradigm for image segmentation that has an encoder and decoder component. The encoder uses convolutional layers and downsampling to extract hierarchical information from input images, while the decoder reconstructs higher-resolution feature maps using upsampling layers. The model has undergone thorough evaluation on the Cityscapes dataset and has demonstrated exceptional performance in picture segmentation, surpassing existing models in terms of accuracy, mean IOU, and mean DICE.

In order to effectively deploy the U-Net concept, it is necessary to follow a series of stages. The tasks involved in this process are data collection and annotation, resizing and standardising images, normalising the data, enhancing the data using augmentation techniques, encrypting masks to convert annotations into categorical masks, applying batch normalisation to stabilise and accelerate training, configuring the U-Net model with max pooling and batch normalisation layers, and applying image preprocessing techniques such as histogram equalisation. Furthermore, it is necessary to divide the dataset into training, validation, and test sets in order to get a fair distribution of classes. The encoder section of the U-Net design utilises convolutional layers, max pooling, and dropout layers to perform downsampling. This process aims to decrease spatial dimensions and capture hierarchical features. The decoder component employs upsampling layers and dropout regularisation to enhance spatial dimensions and integrate high-level and low-level data via skip connections. Utilising batch normalisation in both the encoder and decoder aids in enhancing the quality of the feature maps.

The U-Net model’s performance can be assessed by utilising criteria such as mean IOU, which quantifies the precision of pixel and area categorization. Mean IOU curves for training and validation can offer valuable insights into the model’s capacity to accurately capture object boundaries and spatial relationships. The efficacy and uniformity of the model’s segmentation can be assessed using these metrics. When comparing the performance of other models, the U-Net model demonstrates superior performance in terms of validation accuracy compared to other models such as FCN + Transfer Learning and ENet. The U-Net model demonstrates a validation accuracy of 0.85, showcasing its exceptional capacity to precisely categorise and segment data.

## 7. Conclusion

Semantic segmentation of cityscapes via the use of deep learning is an important and game-changing research topic that offers a more nuanced comprehension of urban landscapes. The implementation of deep learning models solves the complexity and diversity of cityscapes, which unlocks a broad range of applications. Some of these applications include urban planning, transportation management, autonomous driving, and smart city efforts. Semantic segmentation equips decision-makers and stakeholders with the ability to make informed decisions for the purpose of achieving sustainable and effective urban development by offering relevant insights and analyses that are rich in context. In conclusion, this paper has elucidated the utility of the U-Net deep learning model in the realm of Cityscapes image segmentation. The meticulously designed encoder, with its hierarchical feature extraction and downsampling stages, empowers the model with the capacity to grasp intricate spatial context within urban scenes. Batch normalization and dropout layers strategically incorporated in the architecture ensure the model’s robustness against overfitting and expedite convergence during training. The decoder, equipped with upsampling layers and enriched by the inclusion of skip-connections that bridge the gap between low-level and high-level features, facilitates precise segmentation. This synergy between local and global information not only improves the model’s accuracy but also allows it to better capture fine-grained details. Empirical results on the Cityscapes dataset affirm the U-Net’s prowess in semantic segmentation tasks, showcasing its potential to excel in real-world urban scene understanding applications. As urban landscapes continue to evolve, the importance of accurate and efficient image segmentation methodologies cannot be overstated. The U-Net model, as demonstrated in this study, stands as a robust solution for advancing the state of the art in this critical domain.

## Supporting information

S1 Graphical abstract (PNG)
