# Supplementary figures and images for: Semantic segmentation of urban environments: Leveraging U-Net deep learning model for cityscape image analysis
Source: PLoS One. 2024 Apr 5;19(4):e0300767. doi: 10.1371/journal.pone.0300767 (PMC10997063; doi:10.1371/journal.pone.0300767)

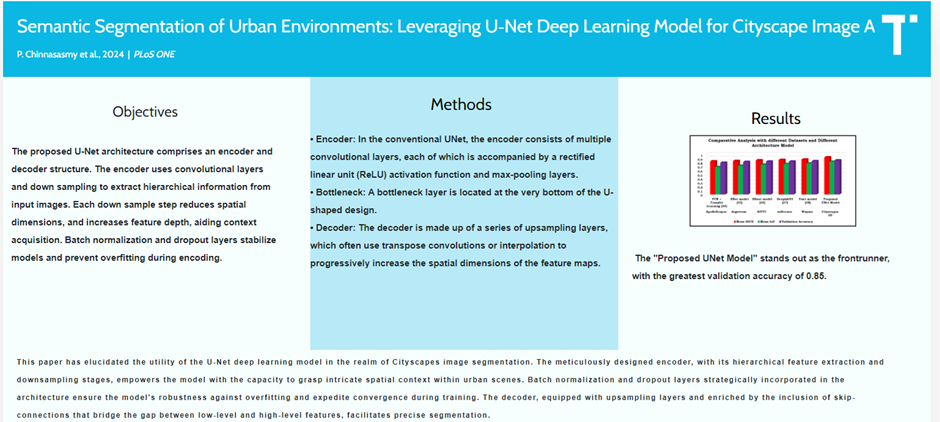

Supplement: S1 Graphical abstract — (PNG) [file pone.0300767.s001.png]
